# Research on Deterministic Figuring of Ultra-Precision Shaft Parts Based on Analysis and Control of Figuring Ability

**DOI:** 10.3390/ma13112458

**Published:** 2020-05-28

**Authors:** Zizhou Sun, Yifan Dai, Hao Hu, Guipeng Tie, Chaoliang Guan, Xuelei Chen

**Affiliations:** 1 College of Intelligent Science and Technology, National University of Defense Technology, Changsha 410073, China; 18302996932@163.com (Z.S.); dyf@nudt.edu.cn (Y.D.); tieguipeng@163.com (G.T.); chlguan@nudt.edu.cn (C.G.); 13308492260@163.com (X.C.); 2 Hunan Key Laboratory of Ultra-Precision Machining Technology, Changsha 410073, China; 3 Laboratory of Science and Technology on Integrated Logistics Support, National University of Defense Technology, Changsha 410073, China

**Keywords:** deterministic figuring, air bearing spindle, ultra-precision, figuring ability, filtering, abrasive belt wear

## Abstract

The application of ultra-precision shaft parts is widely used, such as the spindle core of the air bearing spindle in ultra-precision machine tools. The precision of the spindle core is extremely high, and it is very difficult to obtain directly by traditional Computer Numerical Control (CNC) machine tools but is mostly obtained by manual grinding, whose machining efficiency is greatly limited. Based on the deterministic figuring theory, this paper focuses on the ultra-precision roundness, optimizing the filtering parameters of the measurement error data and studying the generation mechanism of the removal function morphology; the shape of the removal function is adjusted by combining the analysis of the figuring ability and positioning error. Finally, the optimized removal function is used on an experimental steel shaft, the average roundness convergence ratio is 72% higher than that of the original removal function, and the roundness reaches a 0.1 μm level. The result shows that a reasonable filtering of measured data and the removal function adjusted for the surface feature can improve the efficiency and precision of deterministic figuring on shaft parts.

## 1. Introduction

The rapid development of the aerospace industry, precision optics, precision instruments, medical machinery and other fields requires the support of ultra-precision machining technology. As the most important and basic processing equipment in ultra-precision machining, the demand for ultra-precision machine tools is increasing [1]. As one of the core components of ultra precision machine tools, the air bearing spindle has the advantages of small friction resistance, high rotation accuracy, low vibration and noise, and has been widely used in the field of ultra precision machining and measurement [2]. At present, the rotation accuracy of an air flotation spindle can reach 15–25 nm. For example, the rotation accuracy of the SP150 spindle in Precitech’s ultra precision lathe is better than 50 nm. The rotation accuracy of Taylor Hobson’s cylindricity measuring instrument can reach 15 nm. The roundness error (RONt) and the cylindricity error (CYLt) of the journal on the spindle core are the main factors affecting the spindle’s rotation accuracy [3,4]. Therefore, reducing the RONt and CYLt of the spindle core is very important for improving the spindle rotation accuracy of ultra-precision machine tools.

**Figure 1:**
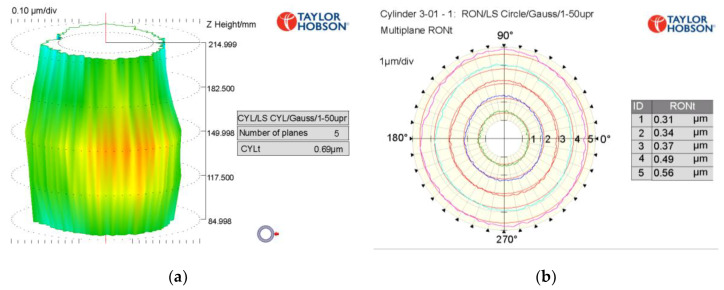
Measurement results of a shaft after ultra-precision cylindrical grinding: (**a**) CYLt; (**b**) Multiplane RONt.

At present, the manufacturing processes of ultra-precision shaft parts mainly consists of turning, grinding and manual grinding, among which various surface treatments are included. After the precision turning of a shaft with a diameter of 100 mm, the RONt can reach 1.5–2 μm, and the CYLt can reach 5–10 μm [5]. The ultra-precision cylindrical grinder, which represents the limit of the precision of traditional cylindrical machining [6,7,8], can reach a RONt of 0.2–0.3 μm and a CYLt of 0.5–0.7 μm; typical machining results are shown in Figure 1. The RONt of the spindle core after manual grinding can reach about 0.1 μm.

Due to the coupling of multiple motion error factors, the accuracy of the workpiece in traditional machining will not be higher than that of the machine tool itself. If we want to further improve its manufacturing precision, the demands for precision on a traditional machine tool spindle are too high and can only be achieved by manual grinding. Manual grinding is non-deterministic machining, and the removal of each grinding process is highly dependent on the experience of the worker, resulting in a low machining efficiency.

Chen [9] et al. innovatively introduced the idea of deterministic figuring to the machining of shaft parts. The principle of deterministic figuring of shaft parts is based on the computer controlled optical surfacing (CCOS) principle [10]. Under the premise of ensuring the linearity and stability of the removal function, calculating the dwell time of the measurement error data by the pulse iteration method, the CNC system controls the dwell time of each position on the surface to realize the quantitative removal of each position, deterministically correcting the shaft’s contour shape. After several phases of machining on a 45# steel shaft with a diameter of 100 mm after turning, the average RONt was reduced from 1.2 μm to 0.4 μm, and the CYLt converged from 6.8 μm to 2.2 μm, reaching the machining accuracy of normal precision cylindrical grinders, verifying the feasibility of the theory and showing its strong potential. However, Chen’s experiments did not deeply study the factors affecting its machining precision. After reaching the above values, the machining precision reached a plateau, and the precision of manual grinding was still not reached.

In order to realize the digital automatic machining of ultra-precision shaft parts, based on the theory of deterministic figuring, this paper starts with the processing of shaft surface error data and the adjustment of the removal function, and studies the relationship between the above two aspects and the RONt of ultra-precision shaft parts, giving the processing method and basis of cylindrical surface error data, analyzing the generation mechanism of the removal function and the adjustment of its morphology, and then realizing the digital ultra-precision RONt figuring of the 0.1 μm level. 

## 2. Data Processing of Measurement Errors

**Figure 2:**
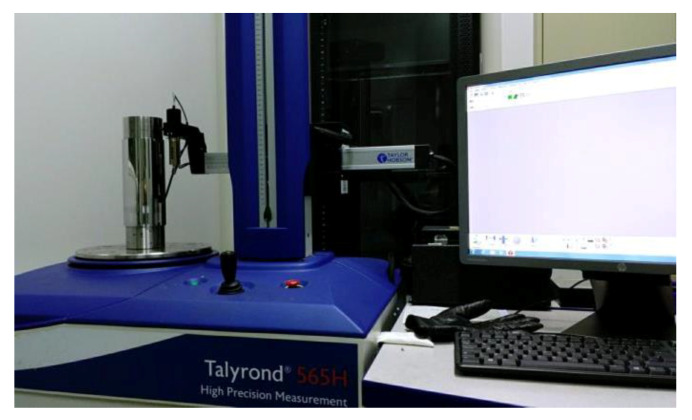
Taylor Hobson’s Talyrond 565H cylindricity measuring instrument.

In the measurement of shaft parts, the contact cylindricity measuring instrument is widely used, and the ruby probe is used to contact the measured surface. The cylindricity measuring instrument we used is Talyrond 565H manufactured by Taylor Hobson (Leicester, UK), the picture of the mesuring instrument is shown in Figure 2, and its main performance parameters are shown in Table 1.

The measured shaft is divided into several sections, and the contour error data of each section are measured respectively, after which the RONt and CYLt are calculated by the algorithm. After machining, the measurement contour of the part always includes the surface roughness contour, waviness contour and the macroscopic shape contour. The surface contour of shaft parts contains different harmonics but is different from the flat workpiece; these harmonics are defined according to the number of undulations per revolution (UPR) [11]. The conversion relationship between the cut-off frequency and the cut-off wavelength of a cylindrical workpiece is given below:

ω_c_ = π*D*/*λ* (1)

where *ω_c_* is the cut-off frequency, *D* is the diameter of the workpiece and *λ* is the cut-off wavelength.

The generation of harmonics is related to the noise during the measurement, the turning accuracy of the machining equipment, the misalignment of the workpiece, the tool vibration and the material characteristics. For traditional shaft machining, the amplitude of harmonics decreases with an increasing frequency.

Because the contact measurement will inevitably produce noise, in order to reduce the impact of noise and fully retain the effective signal, it is necessary to filter out the unwanted signal frequency band by filtering. At present, there is no uniform standard for the parameter selection of filters; therefore, we need to analyze the measurement data and select the filtering parameters that are suitable for the spindle core’s figuring.

### 2.1. Analysis of Measurement Data of the Shaft

**Figure 3:**
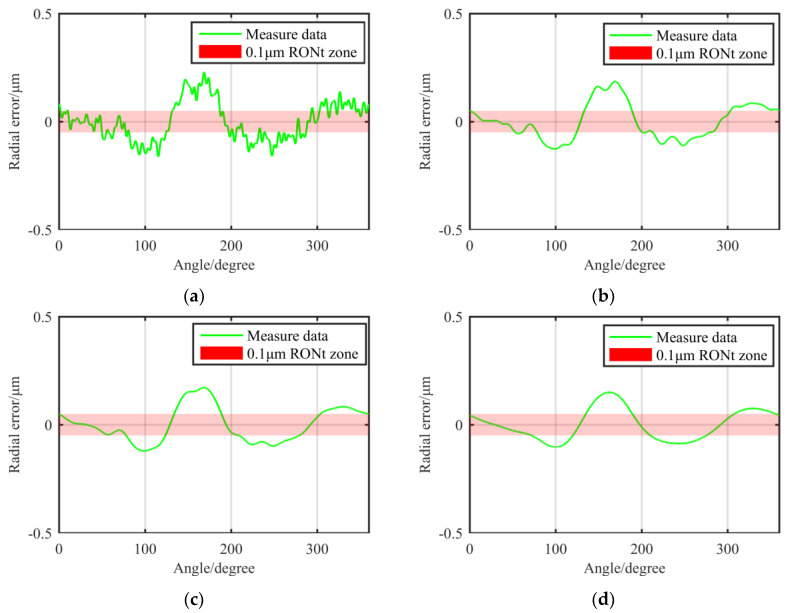
Contour’s error distribution under different filtering parameters:(**a**) 1–50 UPR; (**b**) 1–15 UPR; (**c**) 1–10 UPR; and (**d**) 1–5 UPR.

**Figure 4:**
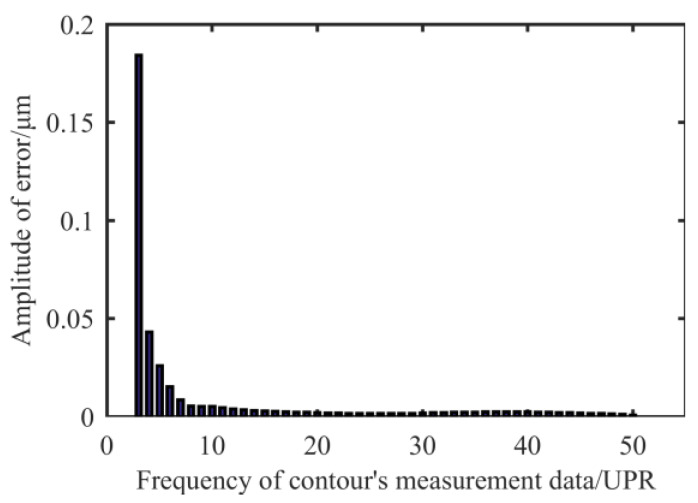
Error’s amplitude corresponding to different frequency components.

We processed the measured contour data of a single section of the surface after grinding, analyzing the factors that affect its RONt to 0.1 μm. The measurement data after different filtering parameters are expanded as follows:

As can be seen from Figure 3, the amplitude of the high frequency error component is small on the shaft surface. As the threshold of the filter becomes smaller, the macro topographical features become more obvious. The contour feature positions that cause RONt to be larger than 0.1 μm are almost in the low frequency range. In terms of the further processing of the measured data, as shown in Figure 4, as the contour frequency increases, the amplitude of error corresponding to this frequency decreases sharply. The amplitude of error at UPR = 10 only accounts for 1.64% of the total RONt. Therefore, we focus on correcting the error component of UPR < 10.

### 2.2. The Requirements of the Spindle’s Working Principle on Figuring

The reason why the rotation accuracy of the SP150 spindle with 0.1 μm RONt can reach 25 nm is due to the homogenization of the gas film supporting the spindle in the bearing. The rotation accuracy of the air bearing spindle can reach 1/4–1/10 of the RONt of the shaft and bearing. When the spindle rotates, the RONt amplitude will affect the distribution of the gas flow field in the gap between the spindle core and the bearing. Due to the uneven flow field inside the gas film, the spindle core will be imbalanced in force, which will cause the spindle movement to shift, and that is how the rotation error occurs. The existence of the gas film has a weakening effect on the fluctuation of the spindle core’s own contour, so that the rotation accuracy can be higher than its own RONt. The homogenization effect of the error is related to the distribution of the circumferential contour error. As the frequency of the contour’s error increases, the distribution of the flow field becomes more uniform and the magnitude of the rotation error decreases.

According to Wang’s [12] research, the rotation error caused by RONt accounts for 46% of the total rotation error value. At the same amplitude, the single undulation has the greatest impact on the rotation error, and the odd undulation has a slightly greater impact than the even undulation. Under the same error amplitude, with the increase of the undulation number (i.e., frequency), the rotation error decreases gradually. The errors that affect the rotation error are mainly concentrated in the low frequency range under 10–15 UPR. Therefore, from the perspective of the working principle of the spindle, we should focus on correcting the low frequency error.

### 2.3. The Requirements of the Actual Machining Conditions on the Shaft Contour

The removal amount of deterministic figuring is formed by the two-dimensional convolution of the removal function and dwell time. The removal function has a long dwell time at the high error point and a short dwell time at the low error point. If the frequency of the cylindrical shape error is high, the dwell time will produce corresponding high-frequency fluctuations, resulting in a high local speed and acceleration, and the dynamic characteristics of the machine tool may not meet the requirements. Taking Chen’s [13] experiment as an example, the program solves that the maximum angular speed of the spindle can reach 6000°/min, while the current maximum speed of the servo spindle of the machine tool is only 3500°/min, which leads to an inaccurate actual removal in certain positions and affects the accuracy of figuring. Secondly, the high-frequency cylindrical surface error results in high requirements for the figuring ability of the removal function, which will cause the positioning error of the actual machining to have a serious impact on the figuring accuracy, and the removal function may even lose its theoretical figuring ability.

Therefore, combined with the analysis of the contour, the working principle of the air bearing spindle and various factors affecting the actual machining, we believe that the 1–10 UPR Gaussian filtering of the measured data of the grinded spindle core can not only guarantee the RONt of 0.1 μm, but also improve the efficiency of the figuring, while reducing the requirements of the actual processing conditions.

In general, the selection of the filtering parameters for the measured data is not fixed in the actual machining. If the filtering threshold is too low, it will result in the loss of part of the surface error information and affect the accuracy of the deterministic figuring. Conversely, an excessively high filtering threshold may contain unnecessary noise, which will cause larger fluctuations in the solution of the dwell time and will also cause greater positioning errors. The selection of the filter parameters is related to the surface quality, figuring ability of the removal function, arrangement of the throttle orifice in the air bearing spindle [14,15,16] and the expected accuracy.

## 3. The Regulation of the Removal Function

### 3.1. Removal Function of Vibrating Abrasive Belt Grinding

**Figure 5:**
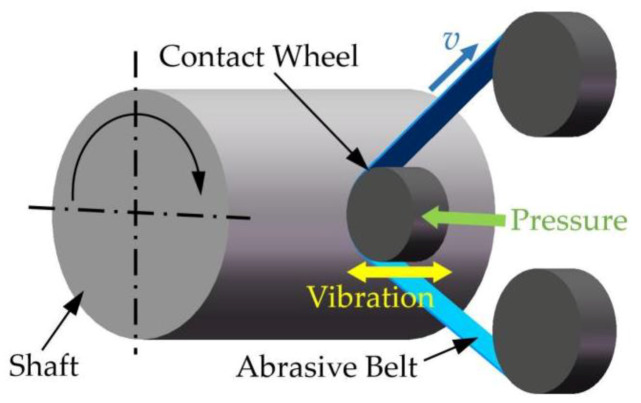
Schematic diagram of abrasive belt deterministic grinding.

As one of the core elements in deterministic figuring, the removal function can be described as the distribution matrix of the material’s removal amount in the machining area under the given machining parameters. In this paper, the form of material removal is shown in Figure 5. The removal function is mainly related to the contact pressure, grit size, belt’s updating speed and vibration frequency. On the premise that the time linearity and long-term stability of the removal function have been verified, a certain amount of material can be removed when the removal function stays at a certain position on the shaft surface for a certain time.

**Figure 6:**
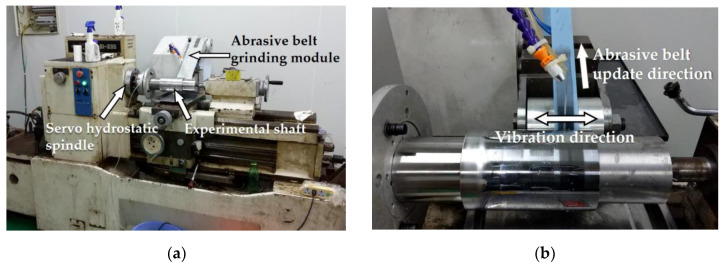
Experimental equipment: (**a**) Modified lathe; (**b**) Vibrating belt grinding module.

As the first generation of machine tools for principle verification, which is shown in Figure 6, we replaced the spindle with a servo hydrostatic spindle on an old lathe. At the same time, the tool holder was replaced by the vibrating belt grinding module on the Z-axis slide, so all positions on the shaft’s surface could be reached under the linkage of the servo spindle and the Z-axis slide controlled by the CNC system.

**Figure 7:**
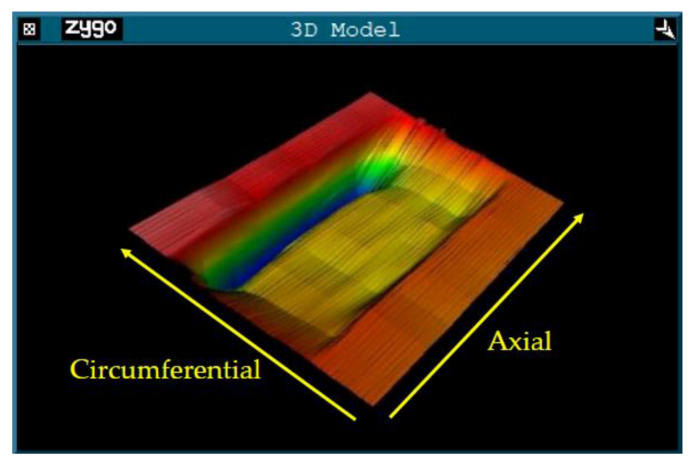
3D morphology of the original removal function extracted from the experiment.

Using the processing parameters shown in Table 2, we obtained the original removal function, whose three-dimensional morphology is shown in Figure 7.

### 3.2. Analysis of the Figuring Ability of the Removal Function

The ability of the deterministic figuring process to correct shape errors depends on features of the removal function. According to Zhou’s [17] research, the figuring ability can be described by the normalized amplitude spectrum of the removal function’s Fourier transform (FT). The figuring ability in one direction corresponds to the normalized FT spectrum of the one-dimensional removal function in the corresponding direction [18]. For the RONt figuring, we should evaluate the ability of the removal function’s shape along the circumference. In actual processing, on the FT spectrum, the cut-off frequency is defined as the corresponding frequency value at 5–10% of the maximum amplitude. Then, the removal function can effectively correct the error’s frequency components in the contour lower than the cut-off frequency. A removal function with a low cut-off frequency usually has a high efficiency in figuring and is suitable for the figuring of macroscopic surface errors with long wavelengths. The removal function with a high cut-off frequency can correct a larger error’s frequency range, but after several phases of figuring, it will cause a medium or high frequency error that is not easy to correct on the surface, and the figuring effect will be greatly affected by the positioning accuracy of the removal function. Therefore, we should choose the removal function reasonably and improve its efficiency on the premise of ensuring the accuracy of the figuring. 

**Figure 8:**
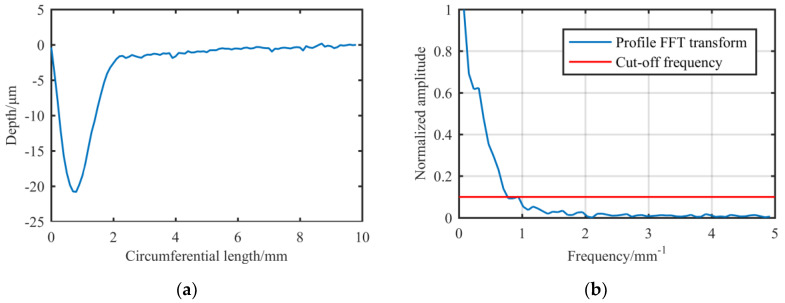
Analysis of the original removal function’s contour: (**a**) Circumferential contour; (**b**) Circumferential contour’s FT spectrum.

The geometrical size of the removal function is a rectangle with a length of about 25 mm and a width of about 10 mm. Its three-dimensional morphology can be decomposed into two contours along the axial and circumferential directions, as shown in Figure 8. This paper mainly focuses on the RONt, so we mainly study the circumferential contour of the removal function. The cut-off frequency is defined as the frequency at 10% of the maximum amplitude, and the cut-off frequency of the circumferential contour is about 0.95 mm^−1^, which can correct the surface error of wavelengths that are longer than 1.05 mm. 

**Figure 9:**
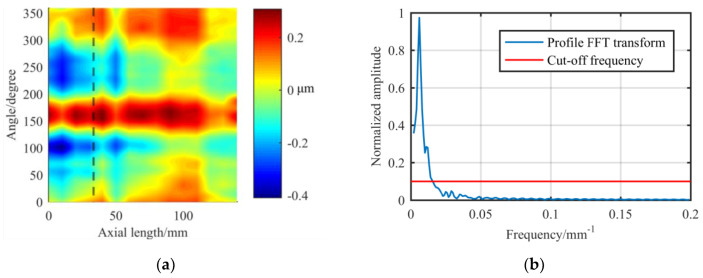
Experimental shaft: (**a**) Expanded surface error data; (**b**) FT spectrum of the circumferential contour which is marked with a dotted line in (**a**).

Then, we analyze the circumferential contour of the experimental shaft. The shaft is made of a 45# steel shaft with a diameter of 100 mm and an effective length of 150 mm. As shown in Figure 9, the cut-off frequency is about 0.015 mm^−1^. Therefore, the original removal function theoretically has the ability to correct the cylindrical surface error.

**Figure 10:**
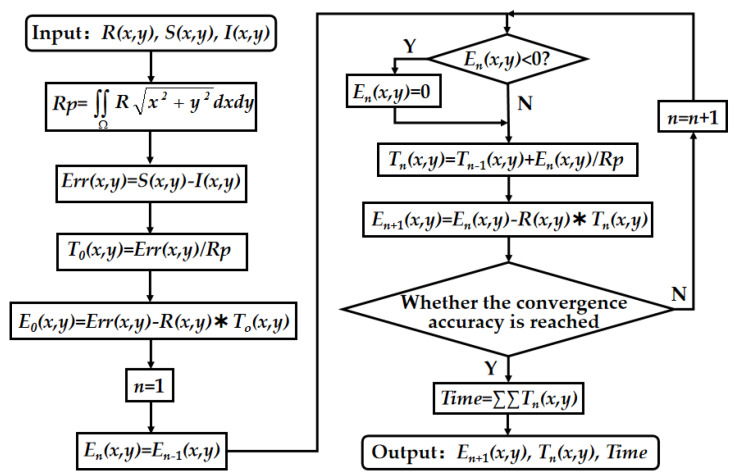
Flow chart of the dwell time’s solution by pulse iteration method.

Next, we use the original removal function to simulate the deterministic figuring of part of the surface in Figure 9. We use the pulse iteration method to calculate the dwell time, and the flow chart is shown in Figure 10. 

Where *x* and *y* represent the axial and circumferential directions of the expanded cylindrical error, *R(x,y)* represents the matrix of the removal function, *S(x,y)* represents the matrix of the measured surface error, *I(x,y)* represents the matrix of the ideal cylindrical error, *Rp* is a removal pulse which centralizes the removal amount from the removal function in the processing area, Ω is the contact area of the removal function, *Err(x,y)* is the material distribution matrix that needs to be removed, *T_n_(x,y)* is the distribution matrix of the dwell time and *E_n_(x, y)* is the matrix of the residual error. When the final residual error meets the convergence accuracy, the total dwell time *Time* will be obtained by summing the dwell time of each discrete point in the matrix *T_n_(x,y),* and the final residual error matrix *E_n+1_(x, y)* is displayed.

**Figure 11:**
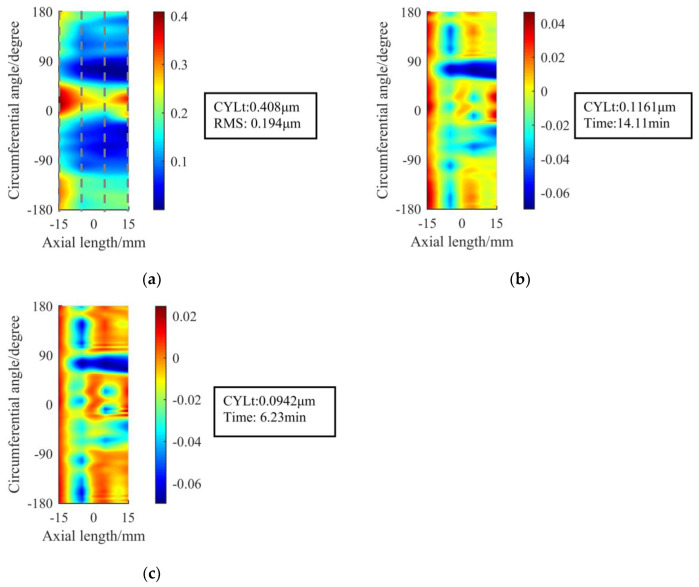
Surface error of the machining simulation by using the original removal function: (**a**) Initial surface with four sections marked with dotted lines; (**b**) Surface after figuring once; (**c**) Surface after the second figuring.

The cylindrical surface error after machining is shown in Figure 11, and the RONt analysis data of the four sections on the machined shaft’s surface is shown in Table 3 (The positions of the four sections are shown in Figure 11a): 

It can be seen from Table 3 and Figure 11 that after 14.11 min of figuring the original removal function successfully corrects the RONt of the three sections below 0.1 μm, and that the RONt of all sections has been successfully converged to within 0.1 μm after the second figuring, which takes another 6.23 min.

### 3.3. Influence of the Removal Function on the Positioning Error

**Figure 12:**
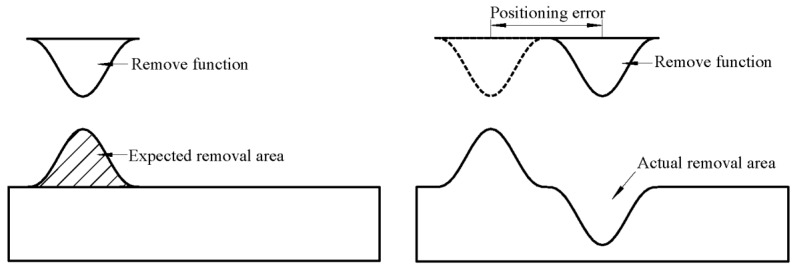
Schematic diagram of the positioning error in deterministic figuring.

It can be seen from the deterministic figuring principle that each position on the surface has a corresponding dwell time after the dwell time solution of the surface error. As can be seen from Figure 12, because the deviation between the starting point in the actual machining and the starting point in the program is unavoidable, due to the existence of the positioning error, the position that should have been removed has actually not been removed, resulting in an unexpected material removal, which affects the results [19,20]. Therefore, we need to analyze the influence caused by the positioning error of the removal function on the ultra-precision RONt figuring.

When a phenomenon similar to the one in Figure 12 occurs, the removal function loses its theoretical figuring ability, which not only results in the surface accuracy not reaching the goal, but also in the contour of the removal function being copied on the machined surface, so that the frequency distribution of the surface error gradually generates high-frequency components.

The positioning error is related to the machined surface and the removal function. For the current experimental conditions, the circumferential positioning error does not exceed 1–2°. Thus, we analyze the positioning error of the removal function in the circumferential direction and make the removal function deviate from the theoretical processing starting point in the circumferential direction by −2°, −1°, 1° and 2°, respectively. The RONt values of the four sections are obtained by simulated machining, and the data is shown in Table 4. 

One disadvantage of deterministic figuring, when compared with traditional turning and grinding, is that during the RONt’s figuring, the positioning error in the circumferential direction will affect the result of figuring, while traditional turning and grinding has no positioning requirement in the circumferential direction. For the shaft with a diameter of 100 mm, the discrete interval of our data in the circumferential direction is 1°, and the discrete interval in the circumferential direction will be adjusted appropriately when the diameter of the shaft changes. The influence caused by the positioning error is related to the discrete interval in the circumferential direction and the variation ratio of the dwell time between the adjacent discrete points.

Based on the figuring principle, when the cut-off frequency of both the shaft’s surface and the removal function are low, the influence of the positioning error is relatively small; when the cut-off frequency of the shaft surface is low and the frequency of the removal function is high, the influence of the positioning error in the first machining is small, but in the subsequent machining the cut-off frequency of the shaft surface will gradually increase and the influence of the positioning error will also gradually increase; when the cut-off frequency of both the shaft’s surface and the removal function are high, the positioning error will have a greater influence on the result.

**Figure 13:**
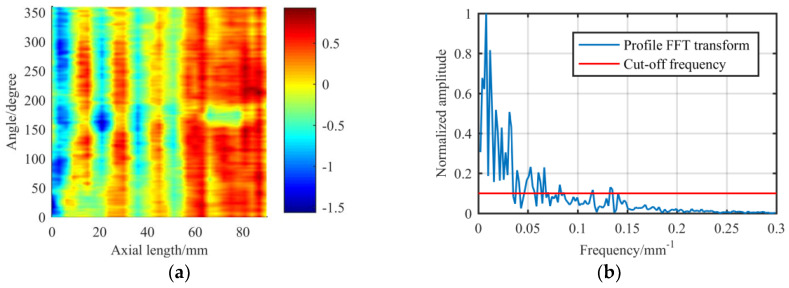
Shaft after several phases of figuring: (**a**) Expanded surface error data; (**b**) FT spectrum of the circumferential contour.

In Chen’s [13] experiments, after several phases of deterministic figuring on the shaft, high-frequency components appear on the circumferential contour, and the cut-off frequency reaches 0.135 mm^−1^, as shown in Figure 13, which is close to nine times bigger than the value of the initial shape. This is also part of the reason why the accuracy of the shaft cannot continue to converge. Thus, the removal function with a lower cut-off frequency will be given priority in order to reduce the influence of practical factors such as the positioning error, on the basis of ensuring the figuring ability, and the shape of the removal function should be adjusted by starting with the mechanism of the removal function.

### 3.4. Generation Mechanism of the Circumferential Contour

According to the Hertz contact theory, when two cylinders with parallel axes make contact, the contact area’s shape is rectangular and the contact pressure along the circumference is theoretically semielliptical [21,22]. The circumferential contour of the original removal function was different from the contact shape of many previous studies on abrasive belt polishing, so we analyzed the cause.

#### 3.4.1. Changes in Stress Distribution Due to Friction Between Contact Surfaces

**Figure 14:**
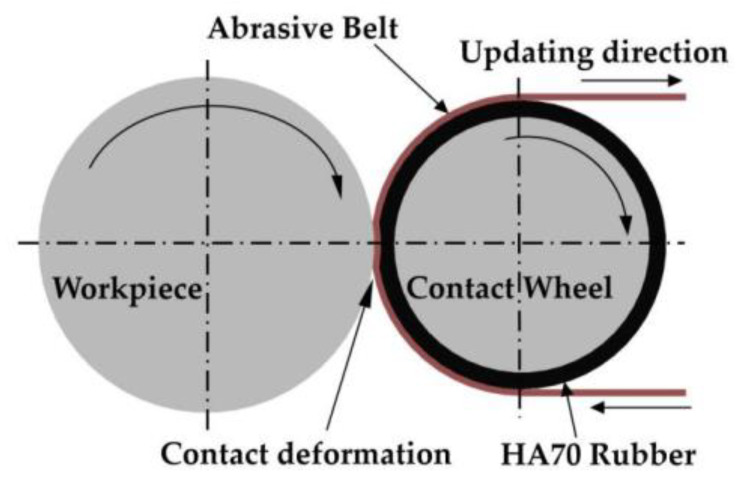
Schematic diagram of the contact between the contact wheel and the workpiece during the deterministic figuring process of the abrasive belt.

**Figure 15:**
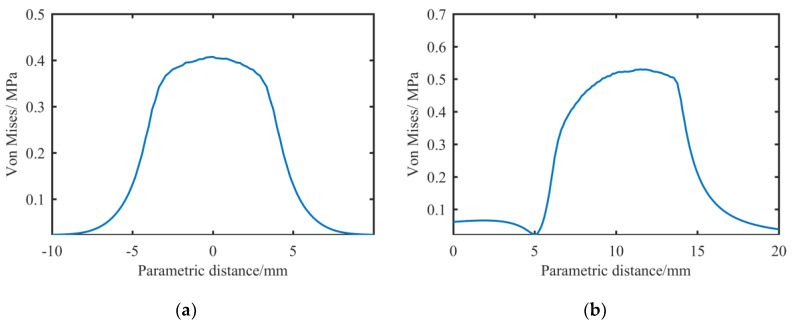
FEA stress simulation results with different friction coefficients in the circumferential direction: (**a**) μ = 0; (**b**) μ = 0.3.

In the actual polishing process, not only is there normal pressure on the contact surface, but there is also a certain friction caused by the relative movement between the abrasive belt, contact wheel and workpiece [23]. The main friction occurs between the abrasive belt and the workpiece, and its dynamic friction coefficient is large; according to Huang’s [24] research, it is between 0.17 and 0.33. The measured value in this experiment equipment is about 0.3. As shown in Figure 14, because the updating speed of the belt is only 1 mm/s in the current experiment, and because the contact wheel can rotate freely, the contact wheel can rotate slowly with the belt. The outer layer of the contact wheel is made of rubber with a hardness of HA70, and it shows a visible deformation during contact. Therefore, considering the influence of friction and deformation, we think that the stress distribution on the contact surface may not be an accurate semiellipse. We combined the friction and the normal force into a direction with equal effect on the shaft for a qualitative analysis, and took two models of μ = 0 and μ = 0.3, respectively, for a finite element analysis. The results are shown in Figure 15:

It can be seen that when μ = 0, the stress distribution is close to the ellipse distribution; when μ = 0.3, the stress distribution is characterized by a steep end and a relatively flat end, which is on the same side as the previous removal function.

#### 3.4.2. Attenuation of the Removal Efficiency Due to Abrasive Belt Wear

**Figure 16:**
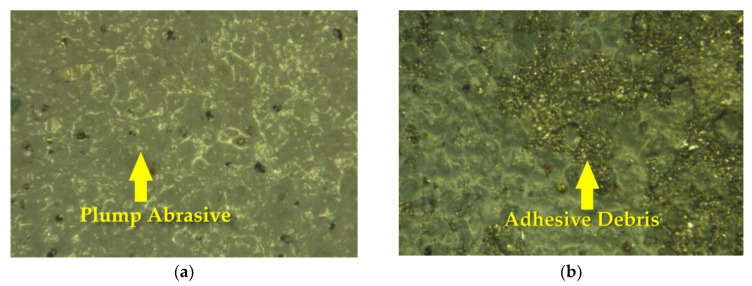
Morphology of the abrasive belt under an Ultra-Depth 3D Microscope: (**a**) Before grinding; (**b**) After grinding.

Compared with the stress change caused by friction, the attenuation of the abrasive belt grinding ability has a greater impact on the shape of the removal function [25]. The wear forms of abrasive belts during the machining process include abrasive grit’s shedding, clogging and blunting [26,27,28], and they all reduce the grinding efficiency. Mulhearn and Samuels [29] concluded that the total mass of material removed (*M_n_*) after *n* passes can be expressed by the following equation:

*M_n_* = *M_∞_*(1 − *e^−βn^*) (2)

where *β* is the deterioration factor, *M_∞_* is the whole weight loss for an abrasive wear test and *n* is the number of passes in the abrasive wear test. They believe this is due to a gradual reduction in the number of effective abrasives by gradually blunting. The blunting rate of abrasive grit increases with the decrease of the grit’s size [30]; when the grit size is small, the clogging of the grit has a greater impact than the deterioration caused by abrasive wear, because the large grits are less likely to adhere to the debris than small ones, and because large ones can break under load to form new sharp abrasives [31]. The surface morphology of the abrasive belt before and after grinding was observed by an Ultra-Depth 3D Microscope. As shown in Figure 16, the abrasive grits on the processed belt are no longer plump, and a lot of iron debris have adhered.

**Figure 17:**
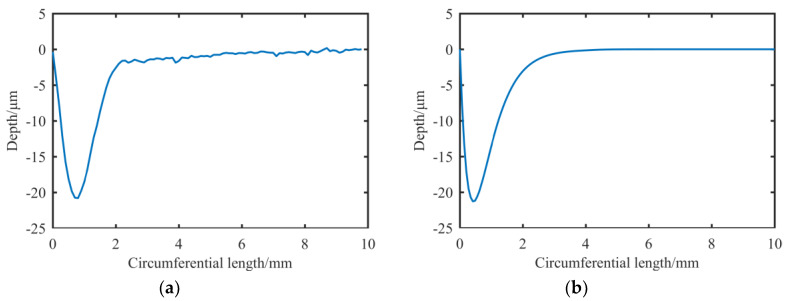
Circumferential contour of the original removal function: (**a**) Actual extraction; (**b**) Simulation generation.

Therefore, we think that another reason for the asymmetry of the shape of the removal function’s circumferential contour is that the updating speed of the belt is slow in previous experiments, which leads to the sharp decline of the belt’s removal efficiency. After calculation, due to the high frequency vibration along the axial direction and the slow updating speed of the abrasive belt, each grit on the abrasive belt passed a total distance of nearly 1200 mm in the grinding area, which makes the abrasive wear obviously be in the contact area. For those experiments which show that the removal profile is symmetrical, the updating speed of the belt is generally 15–30 m/s, which makes the grinding ability of the belt in the grinding area less attenuated. Furthermore, those experiments generally use an abrasive belt with a grit size of 80–600 mesh, while ours is between 1800–3000 mesh, which makes the attenuation of the abrasive belt grinding ability caused by debris blockage more obvious. Finally, we combined the attenuation model of abrasive belt grinding with the previous finite element simulation stress, and obtained an approximate circumferential removal profile. One can see in Figure 17 that the result fits well with the actual removal function.

### 3.5. Analysis of the Optimized Removal Function and its Figuring Effect

**Figure 18:**
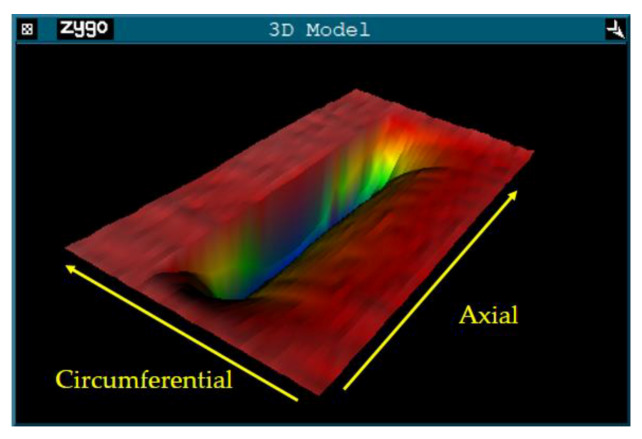
3D morphology of the optimized removal function extracted from the experiment.

**Figure 19:**
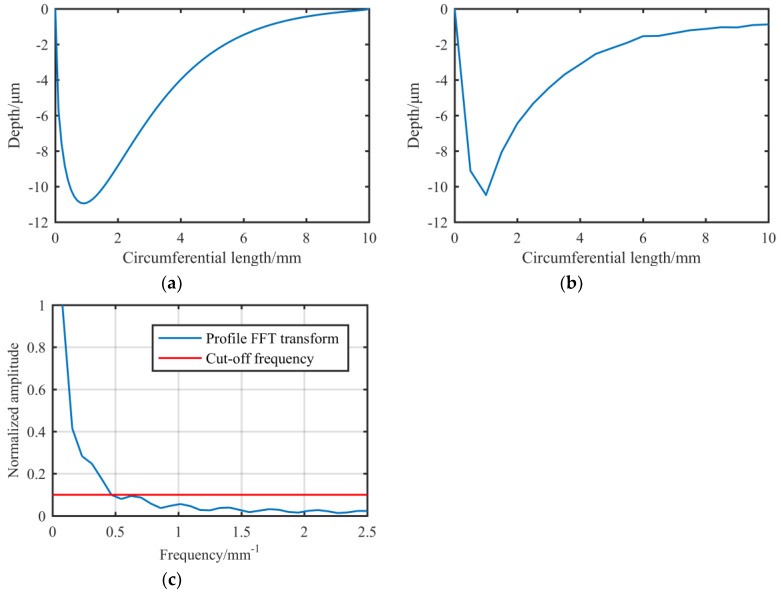
New removal function: (**a**) Generated by simulation; (**b**) Extracted from the experiment; (**c**) FT spectrum of the optimized removal function.

According to the conclusion before, the contour of the removal function can be changed by increasing the updating speed of the abrasive belt, which further reduces the cut-off frequency of the removal function. For this reason, we have replaced a new reducer so that the updating speed of the belt can be up to 10 times faster than the previous speed. The main machining parameters are shown in Table 5. The 3D morphology of the optimized removal function extracted from the experiment using new machining parameters is shown in Figure 18, and the simulated circumferential contour of the new removal function, as well as the experimental extracted circumferential contour of the removal function and its FT spectrum are shown in Figure 19:

The FT analysis shows that the cut-off frequency of the new removal function is about 0.468 mm^−1^, which means that the removal function can also theoretically correct the surface in Figure 11a. At the same time, the contour of the removal function generated by simulation fits the actual extracted contour well, which shows that the analysis of the generation mechanism of the removal function is relatively accurate.

In order to ensure the long-time stability of the removal function, we use open-end abrasive belt polishing. In order to ensure that the abrasive belt will not be exhausted within a limited machining time, the updating speed of the open-end abrasive belt cannot be too high, so a removal contour with an approximate elliptical shape has not been achieved. The updating speed of the new removal function is close to the maximum speed allowed by the experimental conditions. Next, we use the optimized removal function to carry out the simulation figuring experiment.

## 4. Verification Experiment

**Figure 20:**
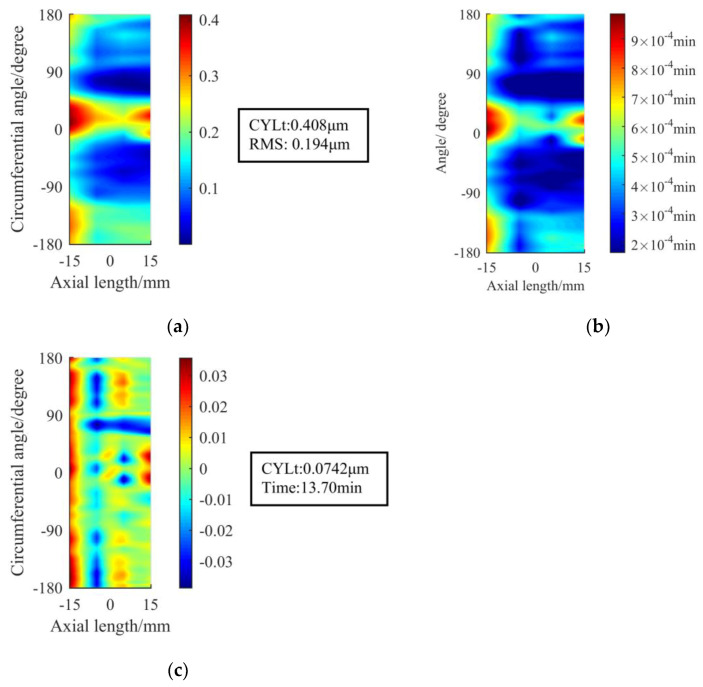
Verification experiment using the optimized removal function: (**a**) The cylindrical surface before figuring; (**b**) The matrix of the dwell time; (**c**) The cylindrical surface after figuring.

**Figure 21:**
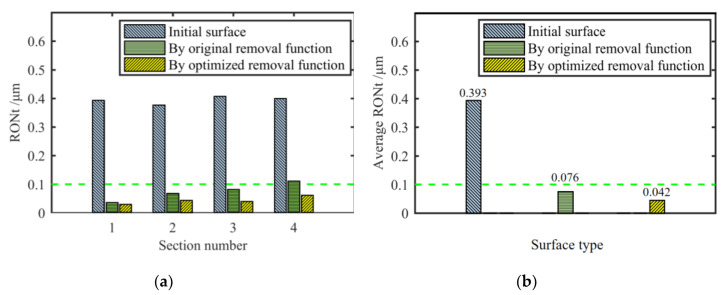
Comparison of RONt after figuring by different removal functions: (**a**) RONt on four sections; (**b**) The average RONt of four sections.

In order to verify the figuring effect of the optimized removal function, we choose the optimized removal function using the parameters in Table 5 to carry out the experiment. According to Figure 20 and Figure 21, after 13.7 min of figuring, the RONt of all four sections of the initial cylindrical surface converges to within 0.1 μm, which shows a better effect and higher efficiency than the original removal function.

**Figure 22:**
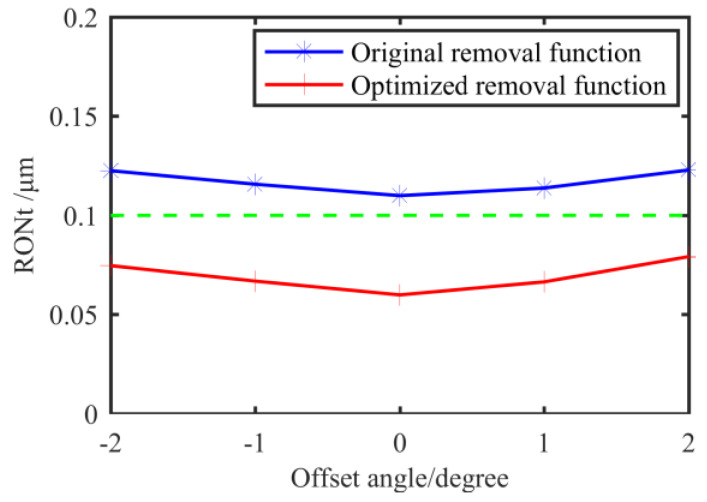
RONt that considering the positioning error on Section 4 after figuring by two removal functions.

Then, we analyze the positioning error. As shown in Figure 22, even if there is a positioning deviation of 2°, the removal function can still ensure that the RONt of all sections be under 0.1 μm, which meets the requirements of the spindle core. For the same surface to be processed, the theoretical average RONt convergence ratio after single figuring using the original removal function is 5.41, while the optimized removal function has reached 9.32, with an increase of 72%. To sum up, the comprehensive figuring effect of the optimized removal function is better in terms of the accuracy, figuring efficiency and the influence of the positioning error.

## 5. Conclusions

- A 0.1 μm level ultra-precision roundness’s figuring of shaft parts can be achieved by using the deterministic grinding of the vibrating abrasive belt.
- The filtering process of the shaft’s surface can improve the efficiency of figuring while ensuring the accuracy. By analyzing the measured data and the working principle of the air bearing spindle reasonably, the corresponding optimized filtering parameters are given.
- Based on the analysis of the figuring ability of the removal function and the surface characteristics of the shaft, the reference basis for optimizing the circumferential contour of the removal function is given.
- By analyzing the micro-morphology and force of the abrasive belt, it can be concluded that the cause of the circumferential contour of the removal function mainly includes the sharp decay of the removal efficiency of the abrasive belt in the grinding area, and the change of the contact stress distribution caused by friction.
- The contour’s prediction model of the removal function is established by combining finite element stress simulation data with the abrasive belt wear model. The contour of the new removal function and the predicted contour of the model are fitted well after changing the machining parameters.
- The circumferential contour of the removal function is optimized by adjusting the updating speed of the abrasive belt, which improves the efficiency and accuracy of the figuring. The roundness convergence ratio during approximately the same machining time is about 72% higher than that of the original removal function. In addition, the wear rate of the abrasive belt can also be changed by changing the contact pressure and vibration frequency to control the morphology of the removal function.
- The results of the cylindrical surface’s figuring in this paper show that the optimized removal function achieves a 0.1 μm roundness of the whole section and can still satisfy the precision even if the circumferential direction has a positioning deviation of 2°.
- Of course, we also need to acknowledge the shortcomings of this method. Firstly, the effect of roundness figuring is affected by the positioning error, which makes demands on the contour along the circumferential direction of the removal function and the shaft; Secondly, as the number of iterative figuring processes increases, higher frequency components with higher amplitudes will appear in the contour of the shaft, and the final accuracy cannot continue to converge. In other words, this method is not suitable for multiple figuring processes on one shaft, and the figuring of the contour error with a large amplitude and high frequency needs further study.

## Figures and Tables

**Table 1:** The main performance parameters of Taylor Hobson’s TR565H cylindricity measuring instrument.

Items	Performance Parameter
Maximum workpiece height	500 mm
Maximum measured diameter	350 mm
Resolution of spindle’s position	0.02°
Available automatic alignment accuracy	<0.8 μm
Available automatic leveling accuracy	<0.8 arc secs
Resolution of the sensor in radial measurement	30 nm
Maximum circumferential sampling accuracy	18,000 points per revolution

**Table 2:** Machining parameters of the original removal function.

Grit Size (μm)	Updating Speed (mm/s)	Vibration Frequency (Hz)	Contact Pressure (MPa)
9	1	6	0.1

**Table 3:** The RONt of four sections before and after figuring by the original removal function.

Surface Type	RONt(1–50 UPR Gaussian Filter)/μm			
	Section 1	Section 2	Section 3	Section 4
Initial surface	0.39086	0.37524	0.40585	0.39864
After figuring once	0.03408	0.06612	0.08024	0.10994
After figuring twice	0.00949	0.06582	0.07197	0.08572

**Table 4:** Relationship between the positioning error of the original removal function and the RONt of each section.

Angle Offset	RONt(1–50 UPR Gaussian Filter)/μm				
	Section 1	Section 2	Section 3	Section 4	Average
2°	0.04872	0.07626	0.08909	0.12288	0.08424
1°	0.03772	0.07110	0.08475	0.11378	0.07684
0°	0.03408	0.06612	0.08024	0.10994	0.07260
−1°	0.03377	0.06148	0.08120	0.11571	0.07304
−2°	0.04331	0.06565	0.08942	0.12253	0.08023

**Table 5:** Machining parameters of the original removal function and optimized removal function.

Type of Removal Function	Updating Speed (mm/s)	Vibration Frequency (Hz)	Contact Pressure (MPa)
Original removal function	1	6	0.1
Optimized removal function	10	6	0.1
